# Network meta-analysis on the efficacy of different interventions for treating thin endometrium

**DOI:** 10.3389/fendo.2025.1575248

**Published:** 2025-08-20

**Authors:** Feng Keng, Wu Ling, Zhang Zhao, Luo Yudi, Li Xiang, Li Derong, Zhong Junjie, Lan Liuping, Zhu Lingling

**Affiliations:** ^1^ Center of Reproductive Medicine, Yulin Maternal and Child Health Hospital, Yulin, China; ^2^ Pediatric Surgery, The People’s Hospital of Guangxi Zhuang Autonomous Region, Nanning, China; ^3^ Qinzhou Maternity and Child Health Care Hospital, Guangxi, China

**Keywords:** thin endometrium, endometrial thickness, clinical pregnancy rate, network meta-analysis, treatment

## Abstract

**Background:**

The incidence of thin endometrium in assisted reproductive technology (ART) is between 1% and 2.5%, yet its treatment options are varied and often show limited efficacy. There is an urgent need to delineate the relative effectiveness of various interventions to guide clinical practice.

**Objective:**

This study aims to systematically compare the efficacy of different interventions for treating thin endometrium via a network meta-analysis, focusing on improvements in endometrial thickness and clinical pregnancy rates.

**Methods:**

A systematic search was conducted in PubMed, The Cochrane Library, EMBASE, CBM, and CNKI databases, covering literature until December 31, 2024. Randomized controlled trials (RCTs) evaluating treatments for thin endometrium were included and assessed for quality using the Cochrane Risk of Bias Tool. Stata 17.0 software was used for the network meta-analysis, employing Bayesian methods to construct network diagrams and calculate the surface under the cumulative ranking curve (SUCRA).

**Results:**

Eighteen RCTs involving six interventions (oral aspirin, Ding Kun Dan, intrauterine infusion of platelet-rich plasma [PRP], intrauterine infusion of granulocyte-macrophage colony-stimulating factor [G-CSF], intramuscular injection of recombinant human growth hormone [rhGH], and neuromuscular electrical stimulation [NMES]) were included. The network meta-analysis revealed: 1) Endometrial thickness: All intervention groups showed varying degrees of effectiveness in increasing thickness compared to the control group. The top three ranked in effectiveness were G-CSF (SUCRA = 78.48%), aspirin (SUCRA = 70.89%), and PRP (SUCRA = 68.14%). 2) Clinical pregnancy rate: PRP ranked highest in improving pregnancy rates (SUCRA = 80.12%), followed by aspirin (SUCRA = 70.29%) and Ding Kun Dan (SUCRA = 62.79%). Overall, PRP showed significant advantages in both increasing endometrial thickness and improving clinical pregnancy rates, making it the most promising intervention.

**Conclusion:**

PRP demonstrates the best clinical application potential in treating thin endometrium, particularly in improving clinical pregnancy rates. Future high-quality RCTs are necessary to further validate and optimize intervention strategies.

## 1 Background

In the successful implementation of assisted reproductive technology (ART), high-quality embryos and adequate endometrial receptivity are two indispensable key factors. Endometrial thickness (EMT) serves as an important morphological indicator for assessing endometrial receptivity, and its clinical significance is widely recognized (1). Currently, there is no consensus on the definition of “thin endometrium,” but most studies consider an EMT of less than 7 mm as thin (2). The pathological mechanisms of thin endometrium have not been fully elucidated. Current evidence suggests that they may be attributed to several factors: reduced expression of estrogen receptors in the endometrium; high vascular resistance in the uterine spiral arteries, resulting in slow glandular epithelial growth; low or absent expression of vascular endothelial growth factor (VEGF) and integrin β3; fibrosis and adhesions leading to impaired angiogenesis in the endometrium; downregulation of metabolic and antioxidant gene expression; and polymorphisms in estrogen receptor genes α and β. These pathological changes result in a significant decrease in endometrial receptivity, subsequently affecting the embryo implantation process (3, 4). The study by Von Wolff et al. indicates patients with EMT ≤ 7 mm have a pregnancy rate of only 7.4%, significantly lower than the 30.8% rate for those with EMT > 7 mm (P = 0.03) (5).This research underscores the importance of optimizing endometrial thickness, as an adequate endometrial thickness facilitates successful embryo implantation.

In clinical practice, the management of thin endometrium remains a significant challenge. Currently, no single therapy has been proven to consistently and significantly improve pregnancy outcomes in this patient population. This situation forces many patients to confront the repeated cancellation of embryo transfer cycles or to endure multiple failures of *in vitro* fertilization and embryo transfer (IVF-ET). Existing treatment strategies primarily encompass the following approaches: Hormonal Therapy: Appropriate supplementation of estrogen (either orally or trans dermally) may be considered; however, its efficacy can vary among individuals, and long-term use may increase the risk of thrombosis. Medications to Improve Endometrial Blood Flow: Agents such as sildenafil (a phosphodiesterase-5 inhibitor), enteric-coated aspirin primarily act by vasodilation to enhance microcirculation within the endometrium, thereby promoting its growth. Platelet-Rich Plasma (PRP) Treatment: Rich in various growth factors, studies suggest that PRP may facilitate endometrial regeneration (6–8). Biologic Therapies: Granulocyte colony-stimulating factor (G-CSF) intrauterine infusion may exert effects by activating endometrial stem cells, although further research is needed to assess its efficacy in patients with severe intrauterine adhesions (9). Physical Therapy: Bioelectric stimulation can improve the condition of the endometrium by modulating local blood flow and neuroendocrine function. Stem Cell Therapy: Preliminary studies indicate that stem cells may repair damaged endometrium via paracrine mechanisms; however, more evidence from well-designed studies is required to validate their efficacy and safety (10, 11). Regarding the selection of treatment protocols, a network meta-analysis conducted by Li et al. compared four commonly used intrauterine infusion regimens. The findings suggest that hCG, along with subcutaneous or intrauterine CSF (SG-CSF), may represent the current optimal choice (12). However, the limitations of this study include a limited number of randomized controlled trials (RCTs) included and insufficient consideration of patient-specific factors affecting treatment efficacy. Therefore, this study aims to incorporate high-quality RCTs of various interventions and employ a network meta-analysis approach to systematically evaluate the relative efficacy of each treatment strategy, with the goal of providing more reliable evidence for accurately tailoring individualized treatment plans.

## 2 Methods

### 2.1 Data and methods

#### 2.1.1 Literature search

A comprehensive search of PubMed, The Cochrane Library, EMBASE, CBM, and CNKI was conducted, with a cut-off date of December 31, 2024. Keywords included “thin endometrium” and “randomized controlled trials,” combining subject headings and free-text terms. Studies were limited to English and Chinese languages, and references were traced for additional relevant studies.

#### 2.1.2 Study inclusion criteria

Inclusion criteria: (i) studies involving patients with thin endometrium; (ii) RCTs with complete data; (iii) outcome measures including endometrial thickness or clinical pregnancy rate; (iv) studies published in English or Chinese.

Exclusion criteria: (i) animal experiments or case reports; (ii) studies with missing or unusable data; (iii) duplicate publications.

#### 2.1.3 Study selection and data extraction

Two researchers independently screened the literature, extracted data, and cross-checked analyses. Initial screening involved reading titles and abstracts to exclude studies not meeting inclusion criteria, followed by full-text review for final inclusion determination.

Risk of bias in included studies was assessed using the Cochrane Risk of Bias Tool, evaluating aspects such as selection (random sequence generation and allocation concealment), performance (blinding of participants and personnel), detection (blinding of outcome assessment), attrition (completeness of outcome data), reporting (selective reporting), and other biases. Each criterion was rated as “low risk,” “high risk,” or “unclear.”

#### 2.1.4 Statistical analysis

RevMan 5.4 software was used for bias assessment. Bayesian network meta-analysis was conducted using Stata 17.0, with effect sizes expressed as standard mean differences (SMD) or odds ratios (OR) and 95% credible intervals (CrI) for statistical significance assessment. SUCRA was used to rank treatment efficacy.

#### 2.1.5 Ethical statement

This study only utilized publicly available data, thus ethical approval was not required. The study was registered with PROSPERO, registration number CRD42025637150.

## 3 Results

### 3.1 Literature search results

**Figure 1:**
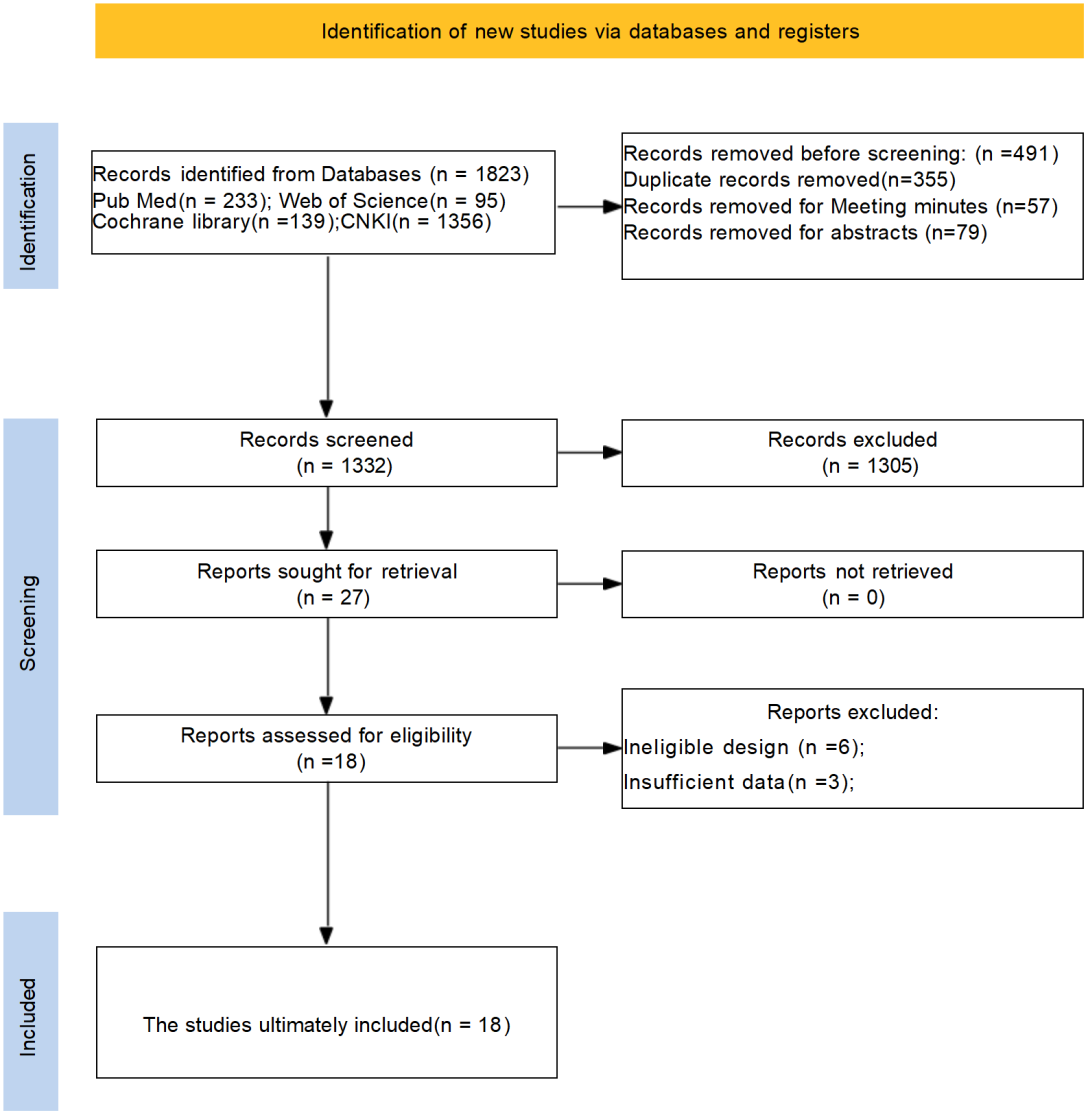
Flowchart and results of literature search.

The PRISMA flow diagram clearly outlines the process of literature screening in this systematic review and meta-analysis, adhering to standardized reporting requirements. An initial search yielded 1823 relevant studies. After screening titles, abstracts, and full texts, duplicate and non-qualifying studies were excluded, resulting in the inclusion of 18 rigorously selected studies, ensuring high quality of the included literature. The literature search process and results are depicted in **Figure 1**.

### 3.2 Characteristics of included studies

Eighteen clinical studies were included, with sample sizes ranging from 28 to 307, totaling 2152 patients. Of these, 1088 participants were randomly assigned to treatment groups, and 1064 to control groups. The studies included 8 on intrauterine PRP infusion (13–20), 4 on intrauterine G-CSF infusion (1, 21–23), 2 on oral aspirin (24, 25), 2 on intramuscular rhGH (26, 27), 1 on oral Ding Kun Dan (28), and 1 on NMES (10), all RCTs. Study characteristics are detailed in **Table 1**.

**Table 1:** General characteristics of included studies.

Study, year	Country	Study design	Group	Sample size	Average age, years	No of embryo transplanted	Intervention	Outcomes
Bodombossou-Djobo et al.2011 (10)	China	RCT	NMES	20	29.75 ± 3.95	1.85 ± 0.49	NMES therapy for 3 to 4 times consecutively (qd for 20–30 minutes)	ET/CPR
			Control	21	30.62 ± 4.54	2.43 ± 0.75	NO NMES therapy	
Chang Y et al.2019 (13)	China	RCT	PRP	34	34.77 ± 0.75	2.00	intrauterine perfusion with PRP 0.5-1.0 ml on the 10th day and the day when progesterone	ET/CPR/EIR
			Control	30	32.64 ± 1.70	2.00	NO intrauterine perfusion	
Eftekhar M et al.2018 (14)	Iran	RCT	PRP	40	31.90 ± 2.26	2.00± 0.40	intrauterine perfusion with PRP 0.5-1.0 ml on the 13th day of HRT cycle	ET/CPR/EIR
			Control	43	32.40 ± 2.63	1.93± 0.61	NO intrauterine perfusion	
Hsieh, Y Y et al.2000 (24)	China	RCT	Aspirin	114	33.10 ± 2.80	NA	Received aspirin (100mg/day) from menstrual day 1 through pregnancy test.	ET/CPR
			Control	122	32.4 ± 3.60	NA	NO aspirin	
Jin, Fengyu et al.2023 (28)	China	RCT	DKP	154	30.7 ± 4.09	NA	Received DKP 10.8 g twice a day orally from the fifth day of the menstrual cycle until 14 days after ovulation	ET/CPR
			Control	153	30.90 ± 3.99	NA	NO DKP	
Mao, Xiaoyan et al.2020 (21)	China	RCT	G-CSF	161	37.79 ± 5.44	NA	intrauterine perfusion with G-CSF either completely filled the uterine cavity or up to 5 mL only, whichever was reached first	ET/CPR/EIR
			Control	143	37.03 ± 4.96	NA	NO intrauterine perfusion	
Nazari, Leila et al.2019 (15)	Iran	RCT	PRP	30	33.90 ± 2.76	NA	intrauterine perfusion with PRP 1.0 ml on day 11–12 in intervention	ET/CPR
			Control	30	32.30 ± 4.79	NA	NO intrauterine perfusion	
Pandey, Divya et al.2023 (16)	India	RCT	PRP	59	29.20 ± 1.89	NA	intrauterine perfusion with PRP 1.0 ml on on the day of ovulation trigger	ET/CPR
			Control	58	29.11 ± 1.89	NA	NO intrauterine perfusion	
Weckstein, L N et al.1997 (25)	America	RCT	Aspirin	15	41.80 ± 3.40	NA	Received aspirin (81mg/day) through 9 weeks after ET	ET/CPR
			Control	13	39.40 ± 4.50	NA	NO aspirin	
Xu, Bin et al.2015 (22)	China	RCT	G-CSF	27	31.40 ± 4.00	2.00 ± 0.00	intrauterine perfusion with 300μg of G-CSF	ET/CPR/EIR
			Control	52	32.00 ± 3.90	2.08 ± 0.33	NO intrauterine perfusion	
Xue-Mei, Wang et al.2016 (26)	China	RCT	rhGH	77	31.30 ± 5.00	2.70 ± 0.50	received 4 IU of rhGH daily by subcutaneous injection from day 3 of the menstrual cycle until the day of progesterone injection	ET/CPR/EBF
			Control	77	30.30 ± 4.10	2.70 ± 0.50	NO rhGH	
Shu-min, Hu et al.2019	China	RCT	rhGH	72	32.50 ± 4.10	1.80 ± 0.40	received 5 IU of rhGH daily by subcutaneous injection from day 10 of the menstrual cycle until the day of progesterone injection	ET/CPR/EBF/CCR/MR
			Control	66	32.10 ± 4.50	1.90 ± 0.30	NO rhGH	
Miao,Liu et al.2024 (19)	China	RCT	PRP	60	31.66 ± 3.14	2.00(1,2)	intrauterine perfusion with PRP 1.0 ml on days 9, 11, and 13 of the FET cycle.	ET/CPR/EBF/MR
			Control	60	32.22 ± 3.74	2.00(1,2)	NO intrauterine perfusion	
Shao-rong Xu et al.2024 (23)	China	RCT	G-CSF	51	33 (29-36)	NA	intrauterine perfusion with 150μg of G-CSF	ET/CPR/EBF
			Control	32	32 (29-35)	NA	NO intrauterine perfusion	
Abduljabbar, Hassan S et al.2022 (17)	saudi arabia	RCT	PRP	35	35.90 ± 4.49	2.11 ± 0.83	intrauterine perfusion with PRP 0.5 ml	ET/CPR
			Control	35	34.60 ± 4.26	2.20 ± 0.80	NO intrauterine perfusion	
Sarvi, Fatemeh et al.2017 (1)	Iran	RCT	G-CSF	13	31.60 ± 3.80	NA	intrauterine perfusion with 300μg of G-CSF	ET/CPR/EIR
			Control	15	31.20 ± 3.20	NA	NO intrauterine perfusion	
Hui,Cheng et al.2020 (18)	China	RCT	PRP	46	31.35 ± 2.36	2.00 ± 0.73	intrauterine perfusion with PRP 1.0 ml on on the day 13 of the FET cycle.	ET/CPR/EIR
			Control	46	32.21 ± 2.13	2.10 ± 0.62	NO intrauterine perfusion	
Hui-dong,Long et al.2020 (20)	China	RCT	PRP	80	35.30 ± 3.75	1.88 ± 0.33	intrauterine perfusion with PRP 1.0 ml on on the day 8 of the FET cycle.	ET/CPR/EIR
			Control	68	33.3 ± 4.36	1.74 ± 0.45	NO intrauterine perfusion	

### 3.3 Risk of bias assessment

**Figure 2:**
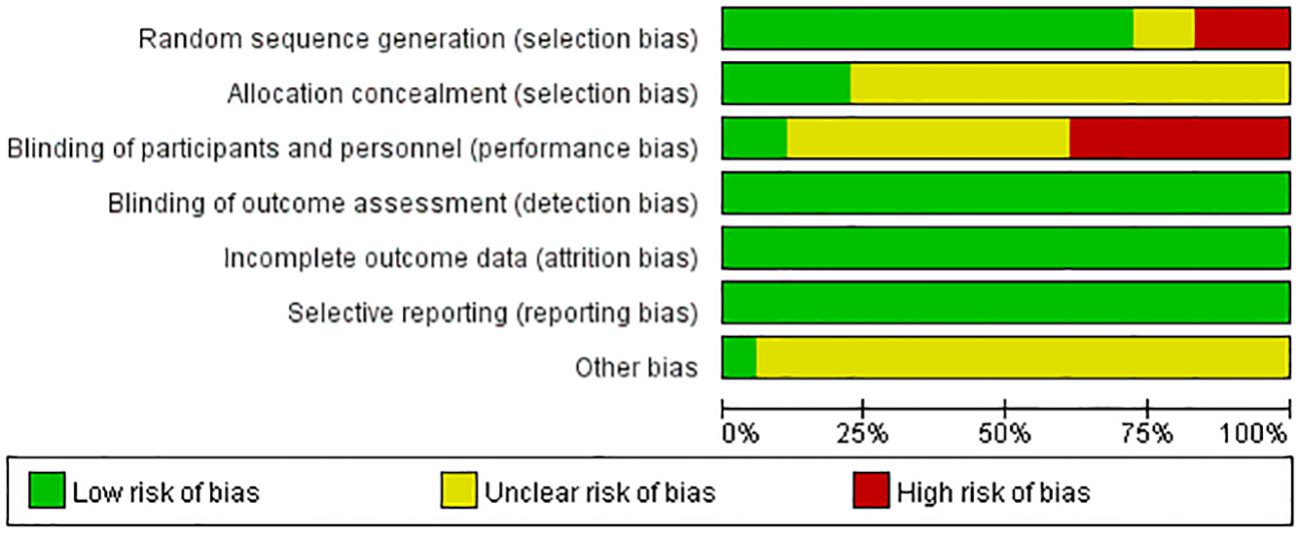
Results of bias risk assessment for all studies.

**Figure 2** displays the risk of bias assessment results for all studies. The quality of included studies was high in terms of random sequence generation and outcome data completeness, but there was a lack of blinding, attributed to the specificity of treatment interventions.

### 3.4 Meta-analysis results

#### 3.4.1 Endometrial thickness results

**Figure 3:**
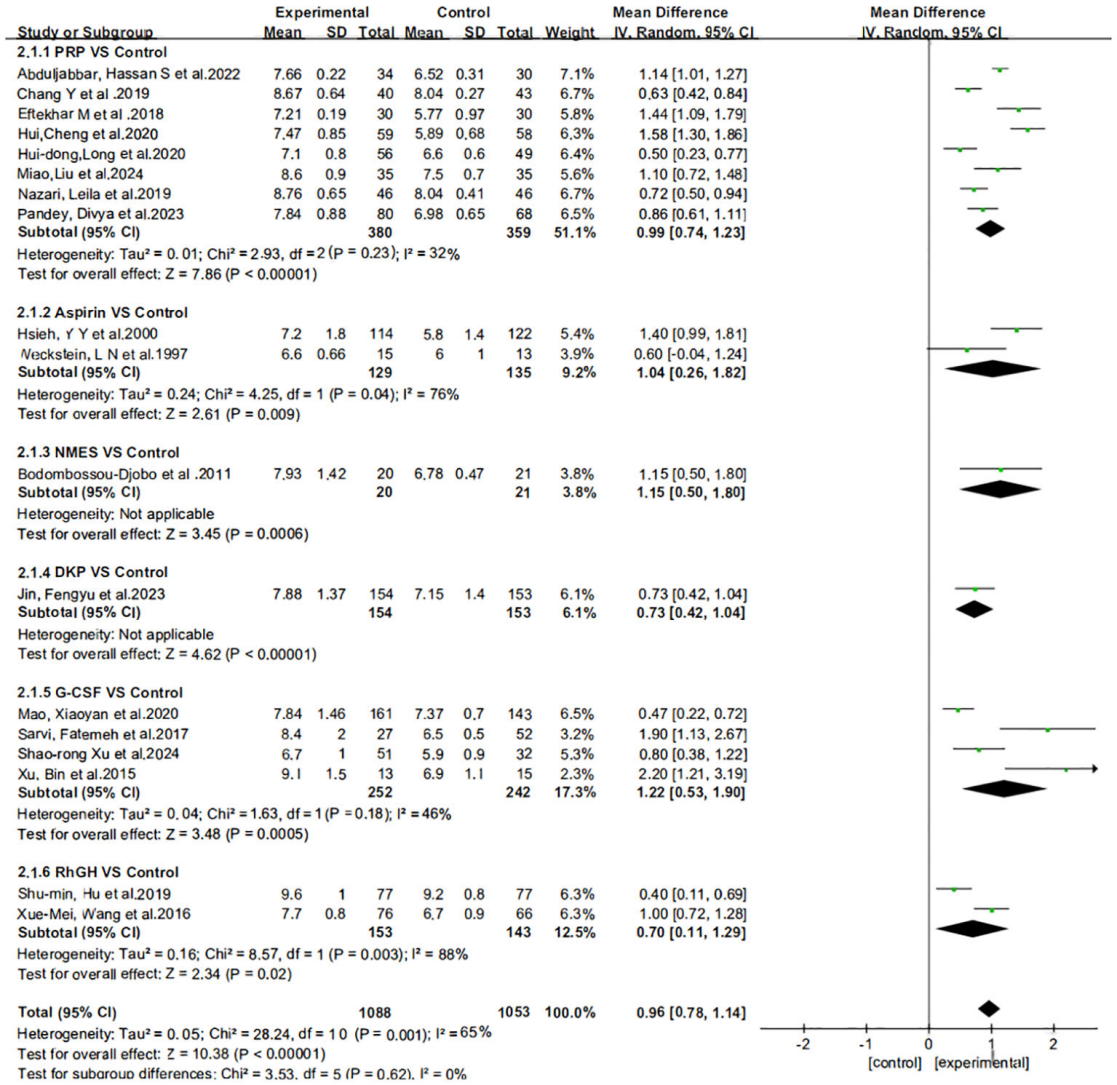
Subgroup analysis and heterogeneity assessment of different treatment regimens on endometrial thickness.

**Figure 3** presents the results of the subgroup analysis on the impact of various treatment regimens on endometrial thickness, illustrated in a forest plot format. The heterogeneity analysis indicates significant variability in the subgroups receiving oral aspirin and intrauterine growth hormone. This heterogeneity may stem from the limited number of included studies. Based on these findings, we employed a random-effects model for subsequent analyses to more accurately estimate the overall effect size.

**Figure 4:**
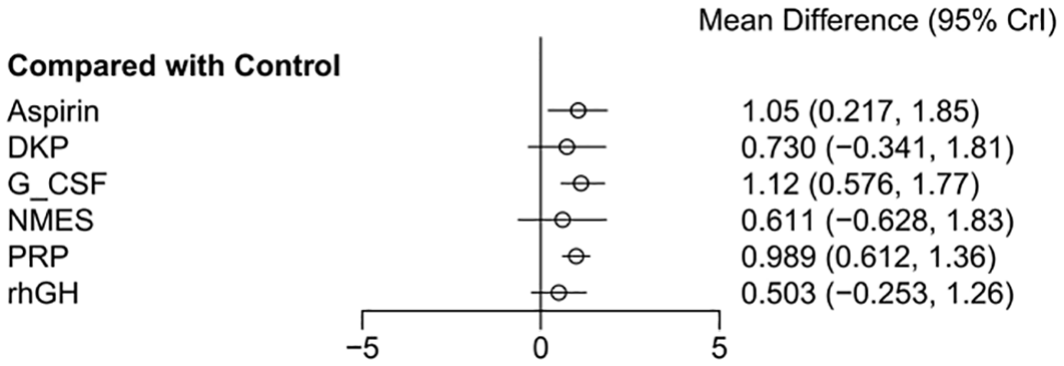
Meta-analysis results of six interventions compared to control group in improving endometrial thickness.

The effect estimates (Mean Difference, MD) for the six interventions (Aspirin, DKP, G-CSF, NMES, PRP, rhGH) relative to the control group in improving endometrial thickness are presented with 95% credible intervals (CrI). Aspirin vs. Control: MD = 1.05, 95% CrI = [0.217, 1.85]. DKP vs. Control: MD = 0.73, 95% CrI = [−0.341, 1.81]. G-CSF vs. Control: MD = 1.12, 95% CrI = [0.576, 1.77]. NMES vs. Control: MD = 0.611, 95% CrI = [−0.628, 1.83]. PRP vs. Control: MD = 0.989, 95% CrI = [0.612, 1.36]. rhGH vs. Control: MD = 0.503, 95% CrI = [−0.253, 1.26]. Summary: Aspirin, G-CSF, and PRP showed statistically significant positive effects. DKP, NMES, and rhGH did not show statistically significant effects, as their credible intervals crossed zero, as shown in **Figure 4**.

#### 3.4.2 Clinical pregnancy rate results

**Figure 5:**
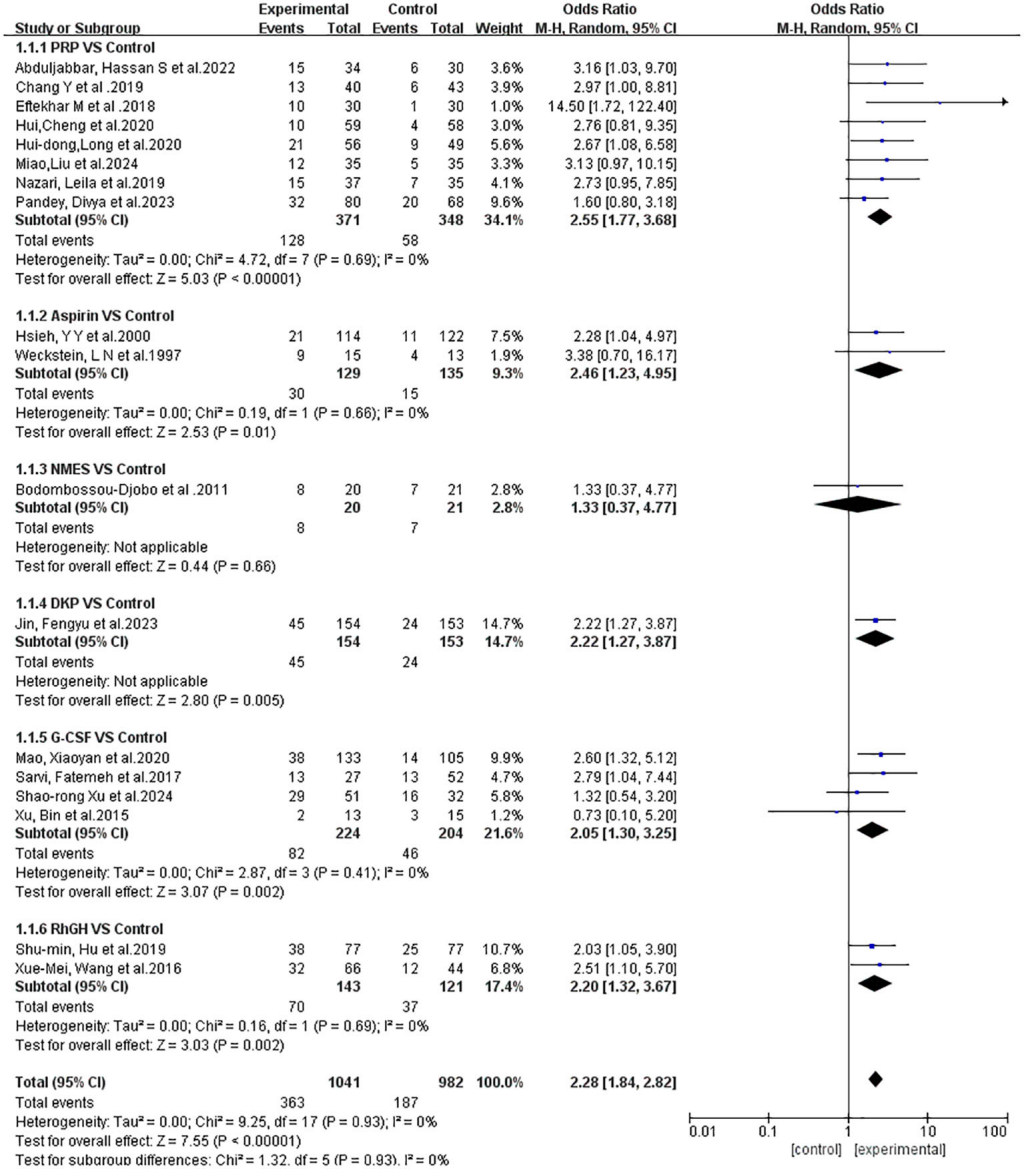
Subgroup analysis and heterogeneity evaluation of various treatment approaches on clinical pregnancy outcomes.

**Figure 5** displays the results of the subgroup analysis regarding the clinical pregnancy outcomes associated with different treatment approaches. The heterogeneity analysis reveals low variability among the studies. Given the low level of heterogeneity detected, we ultimately opted for a fixed-effects model for the analysis.

**Figure 6:**
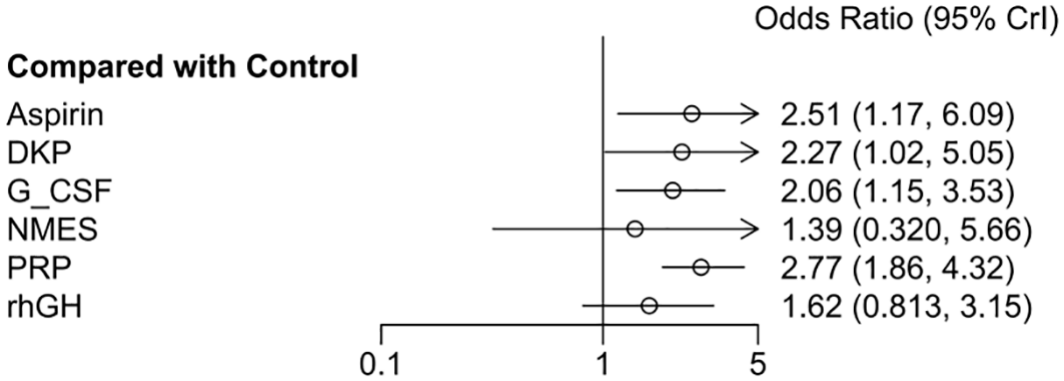
Meta-analysis results of six interventions compared to control group in increasing clinical pregnancy rate.

The odds ratios (OR) for the six interventions (Aspirin, DKP, G-CSF, NMES, PRP, rhGH) relative to the control group in improving clinical pregnancy rates are presented with 95% credible intervals (CrI). Aspirin vs. Control: OR = 2.51, 95% CrI = [1.17, 6.09]. DKP vs. Control: OR = 2.27, 95% CrI = [1.02, 5.06]. G-CSF vs. Control: OR = 2.06, 95% CrI = [1.15, 3.53]. NMES vs. Control: OR = 1.39, 95% CrI = [0.350, 5.66]. PRP vs. Control: OR = 2.77, 95% CrI = [1.86, 4.32]. rhGH vs. Control: OR = 1.62, 95% CrI = [0.813, 3.15]. Summary: Interventions with significant effects included Aspirin, DKP, G-CSF, and PRP, as their credible intervals did not cross one. NMES and rhGH did not show significant effects, as shown in **Figure 6**.

#### 3.4.3 Network evidence of different interventions

**Figure 7:**
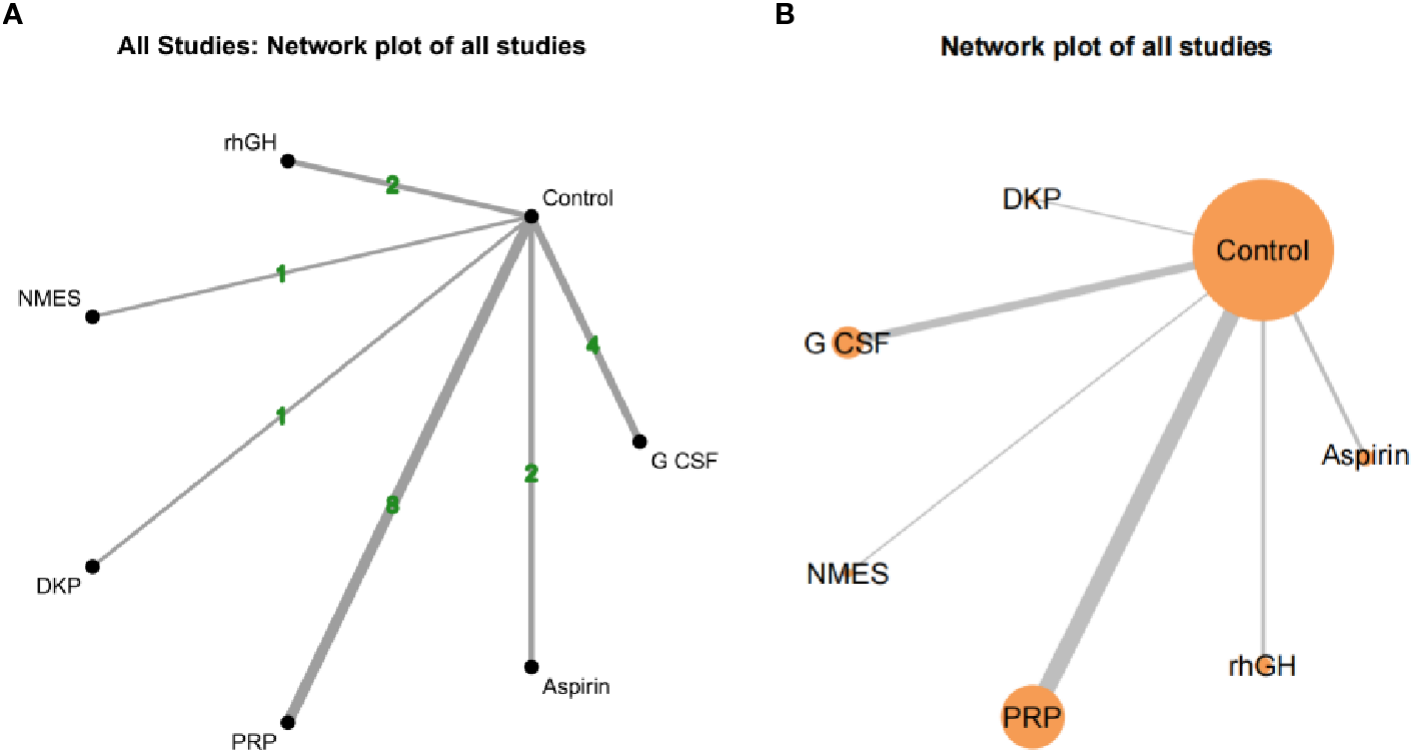
Network evidence for different interventions.

Both network diagrams show the same structure, with Control as the central node directly compared to all interventions. **Figure 7A** emphasizes the number of direct comparisons, while **Figure 7B** highlights the weight of sample sizes and direct evidence. The strongest direct evidence exists between PRP and Control (most studies and largest sample size), leading to more reliable conclusions about PRP’s effectiveness. NMES and DKP have the weakest direct evidence (few studies and small sample sizes), making their results potentially unstable and reliant on indirect evidence credibility. As no closed loops were formed between studies, consistency analysis was not conducted, as shown in **Figure 7**.

#### 3.4.4 Network meta-analysis results

Rows and columns represent different treatment strategies, including Aspirin, Control (placebo), DKP, G-CSF, NMES, PRP, and rhGH. Each cell displays the comparison results between the row and column strategies. Lower left area: comparisons for endometrial thickness. Upper right area: comparisons for clinical pregnancy rate.

Regarding endometrial thickness: Aspirin was significantly better than Control (MD = 0.9, 95% CrI = [0.4, 1.5]), DKP (MD = 1.0, 95% CrI = [0.5, 1.6]), and NMES (MD = 1.2, 95% CrI = [0.6, 1.8]). There was no significant difference between Aspirin and PRP (MD = −0.1, 95% CrI = [−0.6, 0.5]). PRP was significantly better than Control (MD = 1.0, 95% CrI = [0.4, 1.6]) and NMES (MD = 1.2, 95% CrI = [0.6, 1.9]). No significant differences were found between PRP and Aspirin or rhGH. rhGH was significantly better than Control (MD = 1.0, 95% CrI = [0.2, 1.7]) and NMES (MD = 1.3, 95% CrI = [0.5, 2.1]). No significant differences were found between rhGH and Aspirin or PRP.

Regarding clinical pregnancy rate: PRP was significantly better than Control (OR = 2.2, 95% CrI = [1.2, 4.0]), DKP (OR = 2.4, 95% CrI = [1.3, 4.4]), and NMES (OR = 2.9, 95% CrI = [1.5, 5.8]). There was no significant difference between PRP and rhGH (OR = 1.3, 95% CrI = [0.7, 2.6]). rhGH was significantly better than Control (OR = 2.0, 95% CrI = [1.0, 3.9]) and NMES (OR = 2.6, 95% CrI = [1.3, 5.4]). No significant differences were found between rhGH and PRP, Aspirin, or G-CSF. Control was significantly inferior to PRP and rhGH. See **Table 2**.

**Table 2:** Pairwise direct meta-analysis results of study comparisons.

Aspirin	0.4 (0.16, 0.85)	0.9 (0.27, 2.73)	0.82 (0.28, 2.08)	0.55 (0.1, 2.91)	1.1 (0.43, 2.7)	0.64 (0.2, 1.76)
1.05 (0.22, 1.86)	Control	2.27 (1.02, 5.05)	2.06 (1.15, 3.53)	1.39 (0.32, 5.66)	2.77 (1.86, 4.32)	1.62 (0.81, 3.15)
0.32 (-1.04, 1.65)	-0.72 (-1.8, 0.35)	DKP	0.92 (0.33, 2.35)	0.62 (0.12, 3.04)	1.22 (0.5, 3.11)	0.72 (0.25, 2)
-0.08 (-1.18, 0.88)	-1.13 (-1.79, -0.58)	-0.4 (-1.69, 0.77)	G_CSF	0.68 (0.14, 3.1)	1.34 (0.68, 2.81)	0.79 (0.33, 1.85)
0.44 (-1.06, 1.93)	-0.61 (-1.85, 0.63)	0.12 (-1.53, 1.76)	0.53 (-0.81, 1.95)	NMES	1.98 (0.46, 9.46)	1.17 (0.25, 5.62)
0.06 (-0.85, 0.94)	-0.99 (-1.37, -0.62)	-0.26 (-1.39, 0.87)	0.14 (-0.52, 0.9)	-0.38 (-1.68, 0.91)	PRP	0.59 (0.25, 1.25)
0.55 (-0.57, 1.65)	-0.5 (-1.25, 0.25)	0.22 (-1.08, 1.54)	0.63 (-0.28, 1.67)	0.11 (-1.34, 1.57)	0.49 (-0.35, 1.34)	rhGH

Lower left: endometrial thickness data; upper right: clinical pregnancy rate data; numbers in cells indicate comparisons between corresponding row and column treatment strategies. PRP, Platelet-Rich Plasma; G-CSF, Granulocyte colony-stimulating factor; DKP, Ding Kun Dan; rhGH, recombinant human growth hormone.

#### 3.4.5 SUCRA analysis

**Figure 8:**
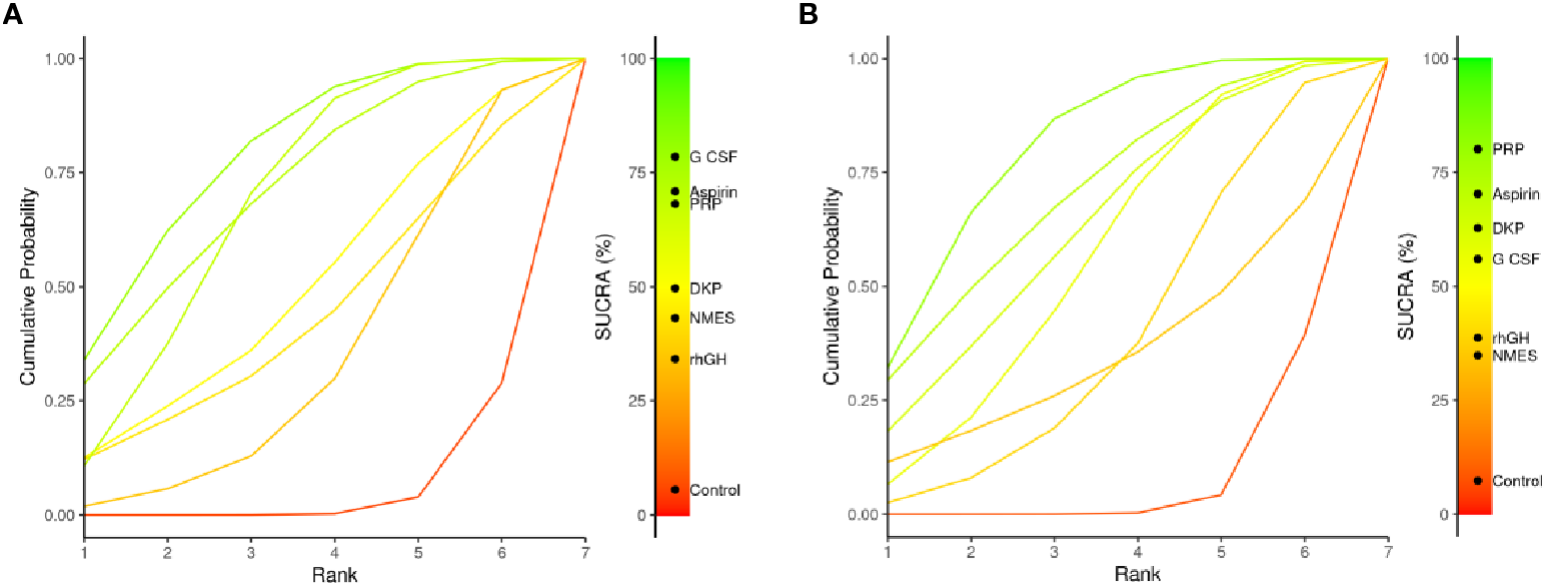
SUCRA probability ranking plot.

The SUCRA plots (Surface Under the Cumulative Ranking Curve) compare the relative ranking probability distribution of multiple interventions for a particular outcome. A curve closer to the top-left corner indicates a higher overall ranking for that intervention in the outcome measure. **Figure 8A** shows endometrial thickness: G-CSF’s curve is notably closer to the top-left corner, indicating it is the best-ranked intervention. Aspirin and PRP also have high SUCRA values and rank as the next best. SUCRA ranking for interventions: G-CSF > Aspirin > PRP > DKP > NMES > rhGH > Control. **Figure 8B** shows clinical pregnancy rate: PRP’s curve is closest to the top-left corner, indicating it is the best-ranked intervention. Aspirin and DKP also have high SUCRA values and rank as the next best. SUCRA ranking for interventions: PRP > Aspirin > DKP > G-CSF > rhGH > NMES > Control. Summary: PRP shows significant advantages in both outcome measures, suggesting it as a comprehensive and effective intervention to be prioritized in clinical practice for treating thin endometrium.

#### 3.4.6 Publication bias

**Figure 9:**
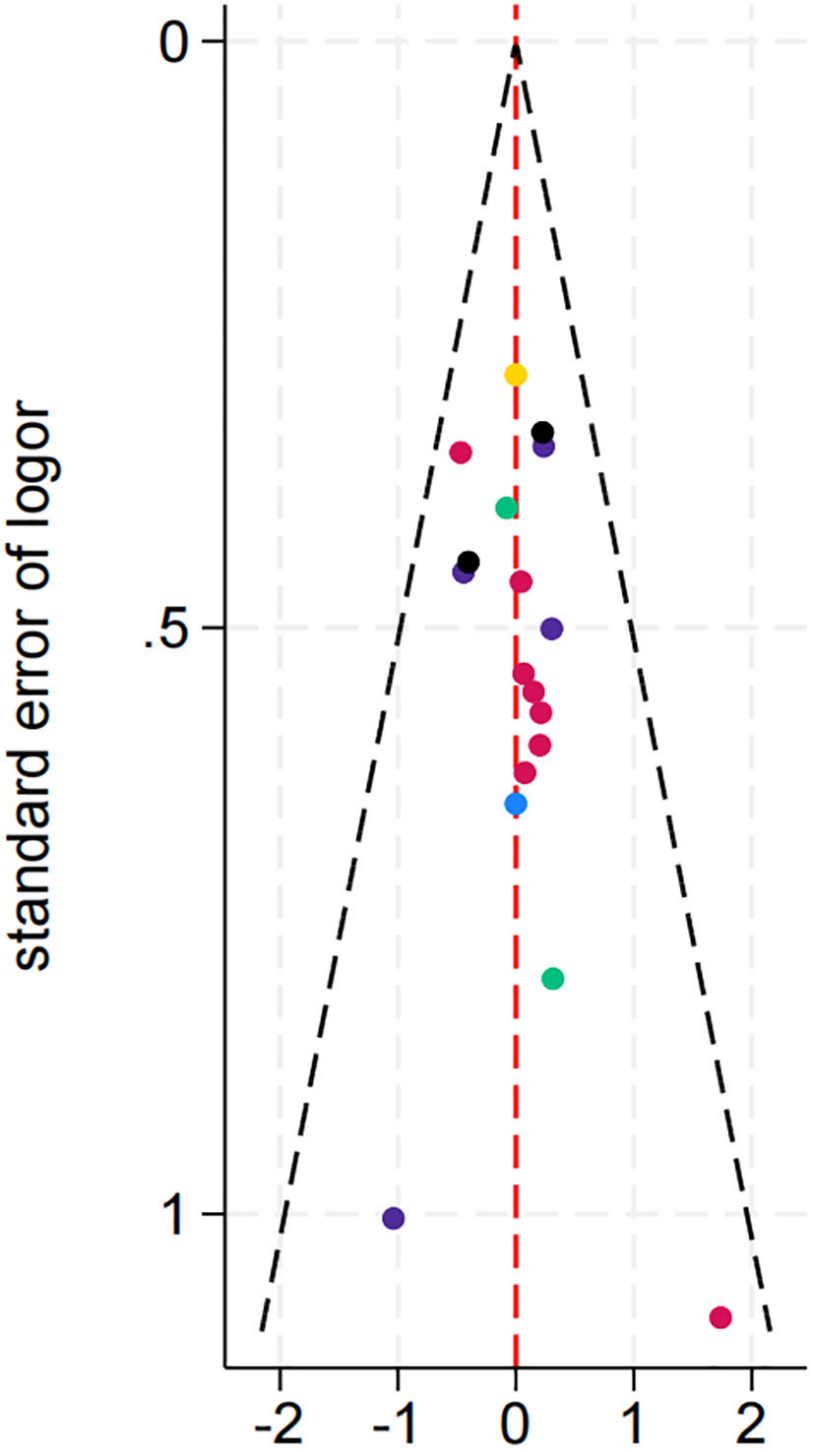
Publication bias plot.

As shown in the **Figure 9**, the effect sizes and standard errors of the studies are symmetrically distributed, suggesting minimal publication bias or other forms of systematic bias.

### 3.5 Discussion

This study incorporated 18 randomized controlled trials (RCTs) that met the inclusion criteria, involving a total of 2,152 participants. While the cumulative probability plots indicated that granulocyte colony-stimulating factor (G-CSF) had a higher SUCRA value for improving endometrial thickness, ranking the interventions as G-CSF > Aspirin > Platelet-Rich Plasma (PRP), direct meta-analysis revealed no significant difference between PRP and Aspirin in terms of enhancing endometrial thickness. However, in direct comparisons and cumulative probability plots for improving clinical pregnancy rates, PRP significantly outperformed other interventions. Thus, although G-CSF may hold an advantage in enhancing endometrial thickness, PRP demonstrates superior efficacy in both improving endometrial thickness and increasing clinical pregnancy rates, suggesting it may be the optimal treatment for patients with thin endometrium.

Despite the variety of treatment options available for thin endometrium, each method has its advantages and disadvantages, and their efficacy can vary significantly. Estrogen primarily acts by activating estrogen receptor pathways, inducing the transformation of endometrial epithelial cells and promoting the mitosis of both endometrial and stromal cells, thereby facilitating the repair of the endometrium. However, the effects of estrogen can differ among individuals, and long-term use may increase the risks of endometrial hyperplasia, thrombosis, and breast cancer (29). Additionally, high doses of estrogen may lead to increased secretion of inflammatory and adhesion factors in the endometrium, which can cause stromal fibrosis and ultimately hinder the repair of the endometrium. Aspirin, as a cyclooxygenase (COX) inhibitor, exerts its effects by inhibiting the synthesis of thromboxane A2, thereby promoting the release of prostacyclin 2 from endothelial cells. This mechanism results in vasodilation, reduced platelet aggregation, and decreased vascular resistance, leading to improved endometrial thickness (EMT) and microcirculation, which enhances endometrial receptivity. However, studies have indicated that COX-2 plays a crucial role in regulating the implantation window of the endometrium and the process of embryo implantation, and the use of aspirin may inhibit COX-2 synthesis, potentially affecting successful embryo implantation (30). Stem cell therapy, which involves primitive undifferentiated cells with self-renewal and differentiation potential, has shown promise in effectively promoting the regeneration and proliferation of the endometrium. Its mechanisms of action primarily include inducing cell differentiation and immune modulation. However, the complexity involved in obtaining and preparing stem cells, along with stringent ethical regulations, poses significant challenges to their clinical application (31, 32). Although granulocyte-colony stimulating factor (G-CSF) has demonstrated some efficacy in clinical treatments, the quality of existing research regarding the dosing and safety of G-CSF is relatively low. Therefore, there is an urgent need for further exploration of optimal dosing regimens and potential risks associated with its use (33, 34).

Platelet-rich plasma (PRP) is a biologic derived from centrifuged autologous peripheral venous blood, rich in platelets and associated cytokines (35). PRP contains 4 to 8 times the platelet concentration found in whole blood and releases various growth factors and cytokines upon activation, including platelet-derived growth factor, transforming growth factor-beta, insulin-like growth factor, epidermal growth factor, fibroblast growth factor, and vascular endothelial growth factor. These autologous growth factors and cytokines promote cell proliferation, differentiation, chemotaxis, and angiogenesis (36–38). During platelet formation, platelets accumulate numerous molecules from the culture medium through endocytosis, including lipid mediators like sphingosine-1-phosphate (SPP), phosphatidic acid, and lysophosphatidic acid, which exhibit anti-apoptotic effects on endothelial cells, participate in chemotaxis, and promote capillary formation. Sphingosine acts as a ligand for endothelial differentiation gene (EDG) receptors, playing a crucial role in angiogenesis, while overexpression of vascular endothelial growth factor receptor 2 sensitizes endothelial cells to vascular endothelial growth factor, initiating angiogenic processes (39, 40). Additionally, PRP can function as an immunomodulator to mitigate immune responses and reduce the release of pro-inflammatory factors such as IL-6, IL-1β, and IL-8 within the endometrium, thereby attracting macrophages and neutrophils (41). Consequently, PRP may enhance endometrial receptivity by modulating the cellular immune microenvironment. In 2015, Chang et al. first applied intrauterine infusion of PRP in frozen embryo transfer (FET) cycles, improving the endometrial conditions of patients with thin endometrium and facilitating successful clinical pregnancies (42). In recent years, studies on PRP treatment for thin endometrium have surged. For instance, Einfeldt et al. conducted a randomized trial showing that PRP injection significantly increased endometrial thickness and local microcirculation, enhancing cell proliferation and angiogenesis to support embryo implantation (43). Additionally, a double-blind randomized study demonstrated higher chemical and clinical pregnancy rates in the PRP group compared to controls, indicating significant efficacy in patients with refractory thin endometrium (15). While PRP improves uterine conditions and pregnancy rates, positioning itself as a new strategy in assisted reproductive technology (ART), it faces clinical application challenges: (i) PRP preparation lacks standardization, and its mechanism of action, optimal concentration, activity, and treatment regimen for different conditions remain unclear; (ii) the relatively low concentration of growth factors necessitates repeated treatments. Further basic research and large-scale, multicenter RCTs are required to validate the long-term efficacy of PRP in ART.

## 4 Study limitations

This study has several limitations: (i)This analysis was restricted to randomized controlled trials (RCTs) published in English or Chinese, and certain treatment options (e.g., Ding Kun Dan and neuromuscular electrical stimulation) had only one eligible study each, which may introduce selection bias and compromise the accuracy and reliability of the findings; (ii)Although data from multiple countries were included, the majority originated from China, potentially limiting the generalizability of the results due to regional variations in patient demographics and clinical practices; (iii) Some of the included studies did not adequately implement randomization or blinding procedures, which may affect the internal validity and robustness of the pooled results.

## 5 Conclusions

In conclusion, the network meta-analysis of the included studies indicates that PRP is the most effective among common treatments for thin endometrium in enhancing endometrial thickness and improving clinical pregnancy rates. However, further high-quality research is needed to substantiate this finding. In clinical practice, we advise careful consideration of these results, with treatment selection based on specific clinical contexts.

## Data Availability

The original contributions presented in the study are included in the article/supplementary material. Further inquiries can be directed to the corresponding author.
